# The Effect of Pediococcus Lactis and Postbiotics on Gut Health and Intestinal Metabolic Profiles

**DOI:** 10.3390/nu18081184

**Published:** 2026-04-09

**Authors:** Jintao Sun, Huaiyu Zhang, Weina Liu, Jinquan Wang, Xiumin Wang, Zhenlong Wang, Hui Tao, Bing Han

**Affiliations:** 1 Key Laboratory of Feed Biotechnology of Ministry of Agriculture and Rural Affairs, Institute of Feed Research, Chinese Academy of Agricultural Sciences, No. 12 Zhong Guan Cun South Street, Haidian District, Beijing 100081, China; 82101235453@caas.cn (J.S.); wangjinquan@caas.cn (J.W.); taohui@caas.cn (H.T.); 2 College of Animal Science and Veterinary Medicine, Shenyang Agricultural University, Shenyang 110866, China

**Keywords:** *Pediococcus lactis*, postbiotics, gut health, microbiota, inflammation, antioxidants

## Abstract

Background: To investigate the effects of probiotics and their postbiotics on mouse health, this study utilized healthy mice randomly assigned to a control group (CK, *n* = 6), a probiotic group (L, *n* = 6, oral gavage 200 μL *Pediococcus lactis*), and a postbiotic group (PL, *n* = 6, oral gavage 200 μL *Pediococcus lactis* postbiotic). Methods: Following 21 days of continuous intervention, changes in gut metabolic profiles, microbial community structure, tissue morphology, and tight junction protein expression were systematically analyzed using metabolomics, 16S rRNA sequencing, hematoxylin and eosin (HE) staining, and immunohistochemistry techniques. Results: The results revealed that screening for significantly altered endogenous metabolites identified core differences concentrated in metabolites related to intestinal barrier repair, anti-inflammation, and antioxidant activity (e.g., 3-indolepropionic acid, astaxanthin, hydroxybenzoic acid). 16S rRNA sequencing revealed that the overall community structure was relatively stable according to principal component analysis, although differences were detected in specific taxa. However, LEfSe analysis identified significantly enriched functional microbial groups at multiple taxonomic levels in the PL group: phylum: Actinomycetota; class: Coriobacteriia; order: Coriobacteriales, Erysipelotrichales; family: Erysipelotrichaceae, Eggerthellaceae; genus: *norank_Erysipelotrichaceae, Intestinimonas*. These results suggest that although the overall community structure remained relatively stable, specific taxa may have differed between groups. Hematoxylin and eosin staining revealed no pathological lesions in intestinal tissues from either group, with intact mucosal architecture. Immunohistochemistry demonstrated significantly elevated expression of intestinal tight junction proteins Claudin 1, MUC-2, Occludin, and ZO-1 in the PL group compared to the CK group (*p* < 0.001). Conclusions: In summary, this probiotic (*Pediococcus lactis*) and its postbiotic showed promising effects, which may be related to changes in specific microbiota taxa, intestinal metabolic profiles, and tight junction protein expression. Beyond maintaining gut microbiota and tissue homeostasis, it enhances intestinal barrier function, suppresses latent inflammation, and boosts antioxidant capacity. Postbiotics may exhibit superior efficacy compared to probiotics. This provides robust experimental evidence for its development and application in gut health products for healthy populations. However, these findings still require further validation in studies with longer intervention periods and in disease models.

## 1. Introduction

*Pediococcus lactis* is a Gram-positive lactic acid bacterium that can metabolize lactic acid and inhibit harmful bacteria. It is commonly isolated from fermented foods and is widely used in the food industry, agriculture, and medicine [1]. Studies have shown that *Pediococcus lactis* has been reported to exert anti-inflammatory effects and regulate lipid metabolism [2]. Research has also found that a combination of *Pediococcus lactis* with *Lactobacillus rhamnosus* and *Lactobacillus rohu* can improve the microbial composition of the vaginal and uterine microbiota in cows post-partum, reduce the incidence of endometritis, and increase pregnancy rates and milk yield [3]. This effect is related to reducing pathogens like *Prevotella* spp., promoting beneficial bacteria such as *Akkermansia muciniphila* [4].

In the field of food fermentation, *Pediococcus lactis* has shown excellent bactericidal and preservative effects, demonstrating potential for biological protection and process assistance. For instance, *Pediococcus lactis* L1 can reduce off-flavors in red sausage storage and improve its color and texture [5]. Additionally, when used together with plant extracts like West Indian cherry and ginger, it produces ham with superior sensory qualities, reduces lipid oxidation during vacuum salting or storage, and maintains a more stable color [6]. Thus, *Pediococcus lactis* shows significant potential in food, both by regulating gut microbiota and inhibiting inflammation to maintain intestinal health and by enhancing its antibacterial and antioxidant properties to extend the shelf life and improve product stability. *Pediococcus lactis* can metabolize tryptophan to produce indole-3-lactic acid. Indole-3-lactic acid, through systemic circulation, crosses the blood–brain barrier and activates the AHR pathway, thereby alleviating neuroinflammation and relieving depression symptoms.

Unlike traditional live probiotics, non-living microorganisms and their derivatives act through immune-metabolic pathways to enhance epithelial barrier function [7], balance gut microbiota imbalance, and regulate oxidative and inflammatory signaling, with no infection risk or survival issues [8]. Postbiotics have similar capabilities to probiotics in changing pathological conditions and preventing diseases [9].

Studies have found that peptidoglycan isolated from Lactobacillus can activate osteoblast differentiation via NOD2 signaling and inhibit osteoclast differentiation, alleviating bone loss in osteoporotic conditions, and has been used as a postbiotic-type therapeutic agent for bone disease treatment [10]. Heat-treated fermented Lactobacillus has been shown in randomized double-blind placebo-controlled trials to significantly increase the secretion of melatonin at night and improve plasma hypothalamic hormone levels during the day, with a greater reduction in nighttime salivary cortisol. These effects help regulate the sleep–wake rhythm, which has potential application for insomnia populations [11]. Research on postbiotics in animals has revealed that they can reduce plasma cholesterol, triglyceride, and LDL levels in broiler chickens [12] while promoting beneficial regulation of the gut microbiota and enhancing short-chain fatty acid production by gut microbes. They are also suitable for dogs suffering from soft stool disorders [13]. Currently, the application potential of postbiotics continues to be explored. Compared with probiotics, postbiotics are not subject to safety constraints associated with live microorganisms and have several advantages, including clearer structural characteristics, no risk of antibiotic resistance transfer, and easier storage, which may facilitate their broader use in pharmaceuticals and food additives. Postbiotics are generally composed of non-purified inactivated microbial preparations containing short-chain fatty acids, exopolysaccharides, bacteriocins, enzymes, and derived peptides. Although research in this field is still at an early stage, increasing evidence suggests that postbiotics have considerable potential to modulate host health. Several postbiotic-based products have been reported to improve gut health by reinforcing the intestinal barrier, alleviating inflammation, and enhancing antimicrobial activity against intestinal pathogens [14]. Nevertheless, as an emerging branch of probiotic research, postbiotics still face substantial challenges in product development and quality control, and robust methods together with rigorous evaluation are required to verify product functionality and consistency [1]. Therefore, identifying the core regulatory mechanisms through which probiotics and postbiotics help maintain gut health may provide theoretical support for substantiating their health benefits and promoting product development.

## 2. Materials and Methods

### 2.1. Experimental Design and Sample Collection

The animal experiment was executed by the Animal Care and Use Committee of the Feed Research Institute of the Chinese Academy of Agricultural Sciences, and was approved by the Ethics Committee of Experimental Animals and inspected by the Feed Research Institute of the Chinese Academy of Agricultural Sciences (IFR-CAAS-20250307, 7 March 2025).

A total of 18 male BALB/c mice were randomly assigned to three groups, with 6 mice in each group. The three groups were: the control group (CK), the live bacteria group (L), and the postbiotic group (PL). After one week of adaptation, 200 μL of 0.9% saline was administered to the CK group. For the L group, *Pediococcus lactis* powder was dissolved in saline and administered via gavage at a concentration of 10^9^ CFU/mL (200 μL). The PL group received heat-inactivated *Pediococcus lactis* inactivated cells (80 °C, 20 min) powder dissolved in saline and administered via gavage at the same concentration (200 μL). The gavage was conducted for three weeks. After the experiment, the mice were anesthetized, blood was collected from the eye, and euthanasia was performed by cervical dislocation. Relevant organs were collected, and colon length was measured. Outcome assessment and data analysis were performed blinded to group allocation.

### 2.2. H&E Staining and Histopathological Observation of Mouse Colon

After collection, colonic tissues from three randomly selected mice in each group (*n* = 3) were dehydrated in graded ethanol and xylene, and then embedded in paraffin using a tissue embedding machine. The paraffin sections were then deparaffinized sequentially using xylene, anhydrous ethanol, and a 75% alcohol gradient, followed by washing with water. The sections were stained with hematoxylin for 3–5 min, rinsed with tap water to remove excess stain, and differentiated with 1% hydrochloric acid. After rinsing, the sections were returned to blue using 0.6–0.7% ammonia water. Dehydration and transparency were performed with ethanol gradients, butanol, and xylene. The sections were mounted with neutral balsam. Microscopic observation was performed to ensure clear contrast between the cell nucleus and cytoplasm with no color interference.

### 2.3. Immunohistochemical Staining of Intestinal Tissues and Average Optical Density (MOD) Analysis

Paraffin sections from colonic tissues of three randomly selected mice per group (*n* = 3) were deparaffinized in xylene, rehydrated through graded ethanol (100%, 95%, and 85%), and washed with distilled water. Antigen retrieval was performed using citric acid buffer (pH 6.0) in a microwave. After cooling, the sections were washed with PBS (pH 7.4). Endogenous peroxidase was blocked by incubating with 3% H_2_O_2_ at room temperature in the dark, followed by washing with PBS. After removal of the blocking solution, the sections were incubated overnight at 4 °C in a humidified chamber with primary antibodies against Occludin (27260-1-AP), ZO-1 (ab221547), MUC-2 (ab272692), and Claudin-1 (28672-1-AP). The next day, the sections were warmed to room temperature, washed with PBS, and incubated with the appropriate HRP-labeled secondary antibody at room temperature for 50 min. After washing with PBS, the sections were visualized using DAB, with the staining time controlled under a microscope. After staining with hematoxylin, differentiation and blue return were performed, followed by dehydration with ethanol gradients and transparency with xylene. The sections were mounted with neutral balsam.

### 2.4. Intestinal Microbiota Analysis (16S rDNA Sequencing)

At the end of the experiment, colonic contents from five mice in each group were collected and sent to Sangon Biotech Co., Ltd. (Shanghai, China) for 16S rRNA gene sequencing. Microbial genomic DNA was extracted using the E.Z.N.A.™ Mag-Bind Soil DNA Kit(Omega Engineering, Inc., Norwalk, CT, USA). The V3–V4 region of the bacterial 16S rRNA gene was amplified using the forward primer 5′-CCTACGGGNGGCWGCAG-3′ and the reverse primer 5′-GACTACHVGGGTATCTAATCC-3′. PCR amplification was performed in two rounds, with universal primers used in the first round and Illumina bridge PCR-compatible primers introduced in the second round. Each sample was amplified in duplicate. The PCR conditions were as follows: initial denaturation at 94 °C for 3 min; 5 cycles of 94 °C for 30 s, 45 °C for 20 s, and 65 °C for 30 s; followed by 20 cycles of 94 °C for 20 s, 55 °C for 20 s, and 72 °C for 30 s; with a final extension at 72 °C for 5 min. PCR products were verified by electrophoresis, library size was assessed by 2% agarose gel electrophoresis, and library concentration was measured using a Qubit 3.0 fluorometer (Thermo Fisher Scientific Inc., Waltham, MA, USA). Equal amounts of each library were pooled for sequencing.

### 2.5. Metabolite Analysis of Mouse Colonic Contents

At the end of the experiment, colonic contents from five mice in each group were collected and sent to Sangon Biotech Co., Ltd. (Shanghai, China) for untargeted metabolomics analysis. Samples were stored at −80 °C and thawed on ice before analysis. Briefly, 20 mg (±1 mg) of each sample was accurately weighed into a centrifuge tube, followed by the addition of 400 μL of 70% methanol extraction solution containing internal standards. The samples were vortexed for 3 min, sonicated in an ice-water bath for 10 min, vortexed again for 1 min, and then incubated at −20 °C for 30 min. After centrifugation at 12,000 rpm and 4 °C for 10 min, 300 μL of the supernatant was transferred to a new centrifuge tube and centrifuged again under the same conditions for 3 min. Finally, 200 μL of the supernatant was transferred into sample vials for LC–MS analysis.

Chromatographic separation was performed on a Waters ACQUITY Premier HSS T3 column (1.8 μm, 2.1 mm × 100 mm) using a mobile phase consisting of 0.1% formic acid in water (A) and acetonitrile (B). The column temperature was maintained at 40 °C, the flow rate was 0.4 mL/min, and the injection volume was 4 μL. Mass spectrometry data were acquired in both positive and negative electrospray ionization modes under instrument-specified conditions.

The raw LC–MS data were converted to mzML format using ProteoWizard (3.0), and peak detection, alignment, and retention time correction were performed using XCMS. Features with missing values in more than 50% of samples were removed. Missing values were imputed using a combined strategy: features with missing values in more than 50% of blank samples were filled with one-fifth of the minimum detected value, whereas those with missing values in less than 50% of blank samples were imputed using the K-nearest neighbor (KNN) method. Data normalization was performed based on internal standards and total signal intensity to reduce technical variation among samples. Batch effects and signal drift were further corrected using quality control (QC)-based normalization when applicable.

Metabolite annotation was conducted by matching the processed features against an in-house database, public spectral libraries, and prediction databases. Only metabolites with a comprehensive annotation score ≥ 0.5 and a coefficient of variation (CV) < 0.3 in QC samples were retained for further analysis. Data acquired in positive and negative ion modes were integrated, and the most reliable metabolite annotation for each feature was retained. Metabolite identification confidence was classified according to the Metabolomics Standards Initiative (MSI), with most annotations considered putative unless confirmed by authentic standards.

### 2.6. Statistical Analysis

The alpha diversity index was determined based on the Shannon index of fecal microbiota. The alpha diversity index is calculated using Mothur (version 1.43.0). beta diversity to assess differences in microbiomes between samples and was often combined with dimensionality reduction methods such as principal coordinate analysis (PCoA) to obtain a visual representation. Differential comparisons are performed using STAMP (version 2.1.3) and LefSe (version 1.1.0) software to identify features that differ significantly in abundance between groups. Correlation coefficients and *p*-values between communities were calculated using Spar CC (version 1.1.0). Evolutionary trees were constructed using Mega (version 7.0.26).

All data are presented as mean ± standard deviation (SD). The normality of data distribution was assessed using the Shapiro–Wilk test, and homogeneity of variances was evaluated using Levene’s test. For comparisons among the three groups (CK, L, and PL), one-way analysis of variance (ANOVA) was performed. All statistical analyses were conducted using GraphPad Prism software (version 8.0.2, GraphPad Software, San Diego, CA, USA). A two-tailed *p* value < 0.05 was considered statistically significant.

## 3. Results

### 3.1. H&E Staining

**Figure 1:**
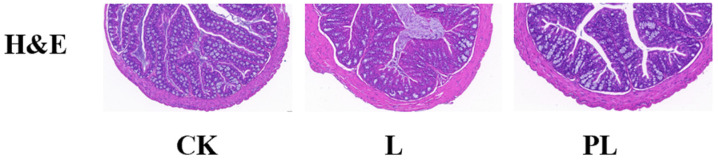
H&E Staining.

Hematoxylin and eosin (H&E) staining results showed that no significant pathological changes were observed in the colonic tissue of both the control and experimental groups of mice. Under the light microscope, the intestinal mucosal morphology was intact in all three groups, with villi arranged neatly and closely. There were no signs of inversion, fracture, or atrophy. The crypt structure was clear and uniform in depth, and the epithelial cell morphology was normal, without degeneration, necrosis, or exfoliation. The lamina propria showed no inflammatory cell infiltration, and the mucosal and serosal layers were intact with no signs of edema, fibrosis, or other abnormalities (Figure 1).

### 3.2. Immunohistochemical Analysis

**Figure 2:**
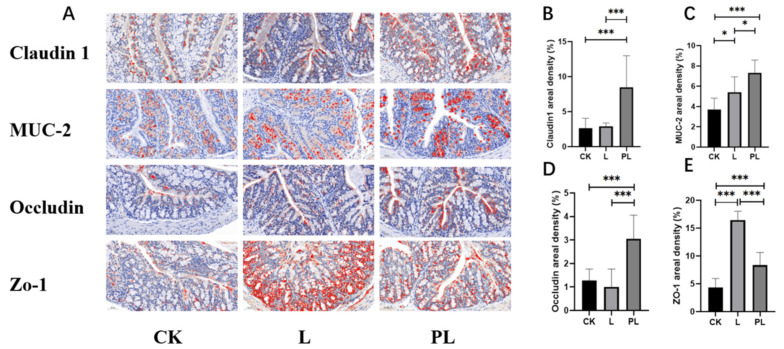
The effects of different groups on the immunohistochemical indices, where a redder color indicates a higher protein content. * means *p* < 0.05; *** means *p* < 0.001. (**A**) Representative images of Claudin 1, MUC-2, Occludin and ZO-1 staining; (**B**) Mucus areal density from Claudin 1 staining; (**C**) Mucus areal density from MUC-2 staining; (**D**) Mucus areal density from Occludin staining; (**E**) Mucus areal density from ZO-1 staining.

Three mice were selected from each group, and three sections were prepared from each mouse. Immunohistochemistry and MOD (mean optical density) quantitative analysis results showed that, compared with the CK group, the expression levels of the tight junction proteins Claudin 1, MUC-2, Occludin, and ZO-1 (Figure 2A) were significantly increased in the PL group (*p* < 0.001). Specifically, the relative expression of Claudin 1 increased by 218%, Occludin by 138%, MUC-2 by 98%, and ZO-1 by 91% (Figure 2B–E).

In the L group, the levels of MUC-2 and ZO-1 were significantly increased, with increases of 46% and 276%, respectively. However, the differences in Claudin 1 and Occludin were not significant. On a molecular level, the intestinal mucosal barrier function in the PL group was superior to that in the L group.

### 3.3. 16S rDNA Sequencing of Mouse Colonic Contents

**Figure 3:**
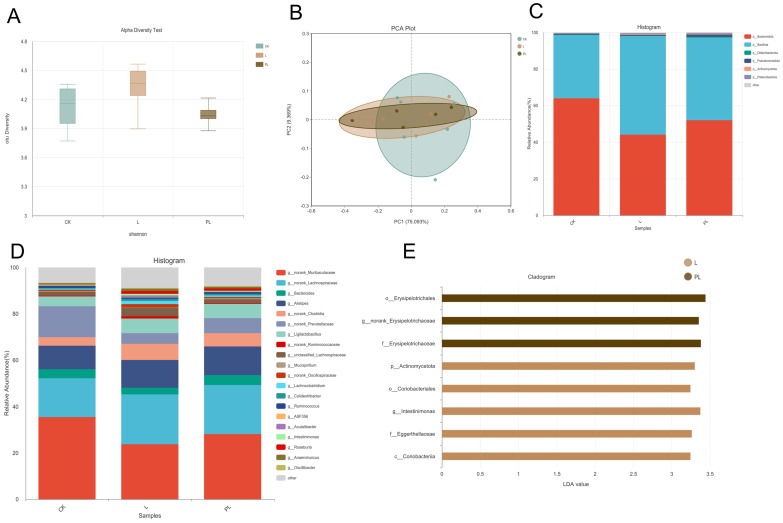
(**A**) Shannon’s indices of the microbial community; (**B**) principal coordinate analysis (PCA) of the genus level; (**C**) The figure shows the abundance of the phylum level of changes in the fecal microbiota. (**D**) The figure shows the genus level of the changes in the fecal microbiota. (**E**) The figure shows the genus LDA score of the two treatments.

16S rDNA sequencing of mouse colonic contents was performed to assess changes in gut microbiota. The results showed no significant change in α-diversity (Figure 3A), as indicated by the Shannon index across all groups. Principal component analysis (PCA) revealed that Principal Component 1 (PC1) and Principal Component 2 (PC2) accounted for 75.093% and 9.369% of the variation in the gut microbiota structure, respectively (Figure 3B). The three groups showed substantial overlap along the PC1 and PC2 axes, with no significant separation, indicating that probiotic intervention did not alter the overall structure of the gut microbiota in healthy mice. At the phylum and genus levels (Figure 3C,D), no significant changes in species abundance were observed between the groups. Further linear discriminant analysis effect size (LEfSe) revealed that, after excluding overall structural differences, there were still significant differences in specific taxa (LDA > 3.0, *p* < 0.05). The PL group exhibited significant enrichment of *Erysipelotrichales*, *Erysipelotrichaceae*, and *norank_Erysipelotrichaceae*, while the L group had increased relative abundance of *Actinomycetota*, *Coriobacteriales*, *Intestinimonas*, and *Eggerthellaceae*. These results suggest that probiotics can selectively enrich specific functional microbiota without disrupting the original gut microbiota structure (Figure 3E).

### 3.4. Metabolomics Analysis of Mouse Colonic Contents

**Figure 4:**
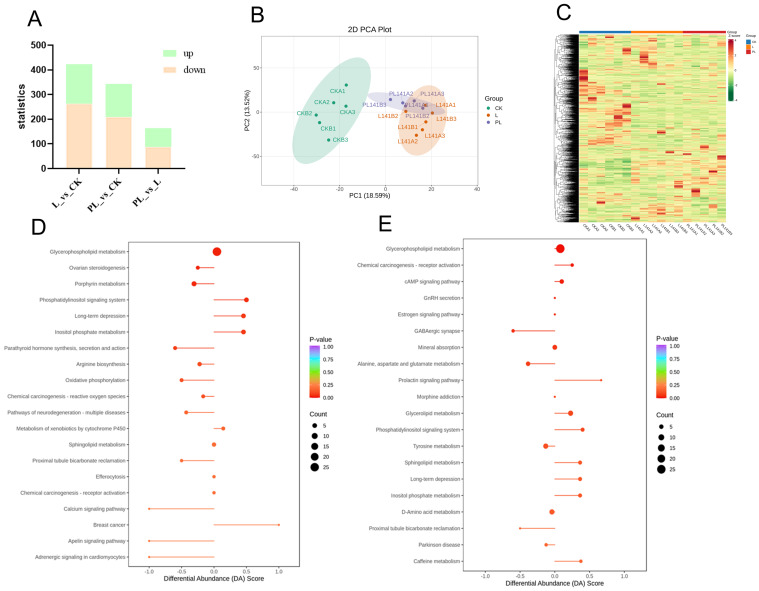
(**A**) Bar chart of differentially abundant metabolites between the L group, PL group, and CK group. (**B**) Principal component analysis (PCA) of the L group and PL group versus the CK group. (**C**) KEGG enrichment analysis of differentially abundant metabolites. (**D**) Overall metabolic pathway changes in the L group and CK group. (**E**) Overall metabolic pathway changes in the PL group and CK group.

The L and PL groups, compared to the CK group, identified 423 and 343 differential metabolites, respectively (Figure 4A). In the L group, 160 metabolites were upregulated and 263 were downregulated. In the PL group, 134 metabolites were upregulated and 209 were downregulated. These metabolites included lipids, amino acids, neurotransmitters, and inflammatory/antioxidant molecules (Figure 4C). We have listed in Table 1 the metabolites that showed significant changes in the L group and the PL group compared to the CK group (VIP > 1, *p* < 0.05).

PCA results showed clear separation between the experimental groups and CK, with overlap between the L and PL groups, indicating significant changes in the intestinal metabolites of mice after probiotic and postbiotic interventions (Figure 4B).

Through KEGG enrichment analysis of differentially expressed metabolites, we observe changes in metabolic pathways. The top 20 pathways ranked by P-value from smallest to largest are displayed. Compared to the CK group, the L group showed upregulation in pathways including Phosphatidylinositol signaling system, Long-term depression, and Inositol phosphate metabolism. Downregulated pathways primarily included Ovarian steroidogenesis, Porphyrin metabolism, Parathyroid hormone synthesis, secretion and action, Arginine biosynthesis, and Oxidative phosphorylation (Figure 4D). KEGG enrichment analysis suggested that the differential metabolites in the PL group, compared with the CK group, may be involved in pathways such as chemical carcinogenesis–receptor activation, the cAMP signaling pathway, and the prolactin signaling pathway, while GABAergic synapse and alanine, aspartate, and glutamate metabolism showed relatively lower enrichment (Figure 4E).

## 4. Discussion

The probiotic potential of *Pediococcus lactis* and its postbiotics relies on its suggested safety and ability to modulate intestinal metabolites through gut microbiota regulation. The probiotic properties and preliminary safety were suggested by examining the pathological status of intestinal sections, the expression levels of tight junction proteins, mucus production, and the changes in gut microbiota and metabolites.

The intestinal barrier is the first line of defense for gut health, and tight junction proteins are the core components of the mechanical barrier in the intestinal mucosa. The integrity of the intestinal tissue and tight junction proteins is crucial to intestinal health [15]. Claudin 1 and Occludin maintain the integrity of intercellular tight junctions [16], while ZO-1 is involved in the assembly and stability of tight junction complexes [17]. MUC-2, the main component of the mucous layer, helps reduce pathogen invasion of the mucosa [18]. The immunohistochemical results are consistent with the fact that the expression of Claudin 1, MUC-2, Occludin, and ZO-1 was significantly increased in the PL group (*p* < 0.001), suggesting that postbiotics significantly enhanced the intestinal mucosal integrity. These proteins, as core components of the intestinal mechanical barrier, are crucial in strengthening the intestinal mucosal barrier. The histological observations further confirmed that both probiotic and postbiotic interventions maintained the normal structure of the intestinal mucosa, with no inflammatory infiltration in the lamina propria and no pathological lesions. Postbiotics, compared to probiotics, showed a stronger protective effect on the intestinal barrier. This phenomenon raises the question of why inactivated preparations outperform live bacteria in a 21-day healthy mouse model. A plausible explanation is that postbiotics provide immediate access to structurally stable microbial components such as peptidoglycans and lipoteichoic acids, which can act as microbe-associated molecular patterns (MAMPs), as well as higher levels of pre-formed effector metabolites. These factors may enable a more rapid or potent activation of host barrier-protective pathways, contributing to the observed superiority of the PL group.

In this study, PCA analysis did not show overall separation of the microbiota between the groups. The microbiota composition remained highly consistent with the physiological state of healthy mice, indicating that probiotics and postbiotics did not cause disruptive microbial restructuring. Instead, they exerted their effects by selectively enriching functional microbiota while maintaining the overall microbial balance. LEfSe analysis identified significant differences in microbiota at multiple taxonomic levels, with *Erysipelotrichaceae* significantly enriched in the PL group, which is known to promote short-chain fatty acid (SCFA) production and maintain epithelial barrier function [19,20,21]. On the other hand, *Eggerthellaceae* and *Intestinimonas* were significantly increased in the L group, known to promote SCFA levels and improve immune function and bone health. These findings highlight that probiotics and postbiotics exert targeted regulation on specific functional microbiota without disrupting the overall gut microbial structure [22,23]. Further enhances the immune homeostasis of the intestinal mucosa, synergizing with the activation of the cAMP signaling pathway [24]. In addition, although the α-diversity and β-diversity analyses indicated that the overall microbial community structure remained relatively stable across groups, the metabolic outputs showed substantial changes. This apparent discrepancy suggests that *Pediococcus lactis*, particularly in its postbiotic form, may modulate the metabolic activity and functional output of the existing microbiota rather than altering its taxonomic composition. Emphasizing this functional modulation provides a more coherent interpretation of how significant metabolite shifts occurred without major structural changes in the gut microbiota.

Metabolomics analysis showed that the intestinal metabolites related to barrier repair, inflammation, and oxidation were significantly altered by the interventions. Key metabolites such as 3-Indolepropionic acid, glutamine, and serine were upregulated, which are involved in intestinal epithelial cell proliferation and repair of damaged mucosal barriers [25,26,27]. The D-amino acid metabolism pathway, upregulated in both the L and PL groups, optimized amino acid homeostasis, supporting the intestinal barrier repair mechanism [28]. Astaxanthin, a potent antioxidant, was significantly upregulated. We speculated that it may be derived from the gut microbiota. It is known to scavenge reactive oxygen species (ROS), inhibit lipid peroxidation, and protect the intestinal epithelium from oxidative damage [29]. Given that *Pediococcus lactis* is not known to synthesize astaxanthin de novo, its elevation may reflect enhanced microbial or host absorption, or metabolic by-products generated during the fermentation process. Similarly, several amino acid-related metabolites may originate from microbial transformation of dietary substrates, as such pathways are commonly influenced by probiotic and postbiotic supplementation. Furthermore, metabolites such as methionine and nicotinamide N-oxide were upregulated, which play important roles in the synthesis of glutathione, a key antioxidant in the gut [30,31]. These findings underscore the dual action of probiotics and postbiotics in enhancing both barrier function and antioxidant defense. This study is limited by its relatively small sample size (*n* = 6 per group), which may reduce the statistical power, particularly for high-dimensional metabolomics analyses.

## 5. Conclusions

This study suggests that Pediococcus lactis, both as a probiotic and postbiotic, may have the potential to promote gut health through effects associated with intestinal barrier function, anti-inflammatory activity, and antioxidant defense. The probiotic properties of *Pediococcus lactis* maintain overall gut microbiota balance, while postbiotics provide enhanced intestinal protection, inflammation inhibition, and oxidative stress reduction. The integrated effects of *Pediococcus lactis* in regulating the intestinal microbiota and metabolites establish a comprehensive “barrier enhancement–anti-inflammation–antioxidant” regulatory system. These findings may support the development of functional foods, particularly for companion animals, where maintaining gut health and improving product stability are important. However, while the findings are promising for gut health maintenance, further studies in disease models (such as IBD or increased intestinal permeability models) are still required to validate these effects.

## Figures and Tables

**Table 1:** Upregulated and Downregulated Metabolites in L and PL Groups Compared to CK Group (VIP > 1, *p* < 0.05).

Metabolite Type	L Group Upregulated Metabolites	L Group Downregulated Metabolites	PL Group Upregulated Metabolites	PL Group Downregulated Metabolites
Lipids	LPC (16:0/0:0), LPC (18:0/0:0), LPC (20:1/0:0)	FFA (16:1), FFA (14:0), FFA (19:1)	LPC (16:0/0:0), LPC (18:0/0:0)	Ceramide-1-phosphate, 13-HODE, Taurocholic acid
Neurotransmitters	Dopamine, Serotonin, 3-Indolepropionic acid	-	Dopamine, Serotonin, 3-Indolepropionic acid	Epinephrine, Norepinephrine
Amino Acids	Methionine, L-Isoleucine, D-Ornithine	GABA, Dimethylglycine	Glutamine, Serine	-
Inflammatory/Antioxidant	Astaxanthin, Methionine, Nicotinamide N-oxide	-	Astaxanthin, Curcumin, 9S-HOTrE	-

## Data Availability

The original contributions presented in this study are included in the article. Further inquiries can be directed to the corresponding author.
